# Waste to Worth: A diagnostic accuracy of Xpert MTB/XDR on contaminated liquid cultures to salvage the detection of drug-resistant tuberculosis

**DOI:** 10.21203/rs.3.rs-6409041/v1

**Published:** 2025-04-12

**Authors:** Yonas Ghebrekristos, Erick Auma, Zama Mahlobo, Rouxjeane Venter, Natalie Beylis, Jay Achar, Brigitta Derendinger, Sarishna Singh, Megan Burger, Christoffel Opperman, Robin Warren, Grant Theron

**Affiliations:** DSI-NRF Centre of Excellence for Biomedical Tuberculosis Research, and SAMRC Centre for Tuberculosis Research, Division of Molecular Biology and Human Genetics,Faculty of Medicine and Health Sciences, Stellenbosch University, Tygerberg, Cape Town, South Africa; DSI-NRF Centre of Excellence for Biomedical Tuberculosis Research, and SAMRC Centre for Tuberculosis Research, Division of Molecular Biology and Human Genetics,Faculty of Medicine and Health Sciences, Stellenbosch University, Tygerberg, Cape Town, South Africa; DSI-NRF Centre of Excellence for Biomedical Tuberculosis Research, and SAMRC Centre for Tuberculosis Research, Division of Molecular Biology and Human Genetics,Faculty of Medicine and Health Sciences, Stellenbosch University, Tygerberg, Cape Town, South Africa; DSI-NRF Centre of Excellence for Biomedical Tuberculosis Research, and SAMRC Centre for Tuberculosis Research, Division of Molecular Biology and Human Genetics,Faculty of Medicine and Health Sciences, Stellenbosch University, Tygerberg, Cape Town, South Africa; Division of Medical Microbiology, Department of Pathology, University of Cape Town, Cape Town, South Africa; DSI-NRF Centre of Excellence for Biomedical Tuberculosis Research, and SAMRC Centre for Tuberculosis Research, Division of Molecular Biology and Human Genetics,Faculty of Medicine and Health Sciences, Stellenbosch University, Tygerberg, Cape Town, South Africa; DSI-NRF Centre of Excellence for Biomedical Tuberculosis Research, and SAMRC Centre for Tuberculosis Research, Division of Molecular Biology and Human Genetics,Faculty of Medicine and Health Sciences, Stellenbosch University, Tygerberg, Cape Town, South Africa; National Health Laboratory Service, Greenpoint Tuberculosis Laboratory, Cape Town, South Africa; DSI-NRF Centre of Excellence for Biomedical Tuberculosis Research, and SAMRC Centre for Tuberculosis Research, Division of Molecular Biology and Human Genetics,Faculty of Medicine and Health Sciences, Stellenbosch University, Tygerberg, Cape Town, South Africa; National Health Laboratory Service, Greenpoint Tuberculosis Laboratory, Cape Town, South Africa; DSI-NRF Centre of Excellence for Biomedical Tuberculosis Research, and SAMRC Centre for Tuberculosis Research, Division of Molecular Biology and Human Genetics,Faculty of Medicine and Health Sciences, Stellenbosch University, Tygerberg, Cape Town, South Africa; DSI-NRF Centre of Excellence for Biomedical Tuberculosis Research, and SAMRC Centre for Tuberculosis Research, Division of Molecular Biology and Human Genetics,Faculty of Medicine and Health Sciences, Stellenbosch University, Tygerberg, Cape Town, South Africa

**Keywords:** Xpert MTB/XDR, contaminated cultures, drug susceptibility testing, tuberculosis

## Abstract

**Introduction:**

Mycobacterium Growth Indicator Tube (MGIT) 960 culture is critical for tuberculosis (TB) drug susceptibility testing (DST) but vulnerable to contamination. We evaluated the accuracy of Xpert MTB/XDR, a molecular DST for isoniazid, fluoroquinolone, amikacin, and ethionamide, on to-be-discarded contaminated growth.

**Methods:**

Xpert MTB/XDR was applied to acid-fast bacilli-negative contaminated cultures from sputum from people with rifampicin-resistant TB when Xpert MTB/XDR on sputum was unsuccessful (not resistant or susceptible for all drugs) 1) at diagnosis (Cohort A) or 2) during treatment monitoring (Cohort B). Future DSTs within three months served as a reference standard. We determined potential care cascade improvements.

**Results:**

In Cohort A, 10% (66/650) of people were culture-contaminated. 89% (59/66) were contaminated growth Xpert MTB/XDR TB-positive. Sensitivity and specificity for isoniazid, fluoroquinolone, amikacin, and ethionamide resistance were 100% [95% confidence interval (CI) 85, 100] and 100% (79, 100), 100% (59, 100) and 100% (89, 100), 100% (16, 100) and 100% (91, 100), and 100% (72, 100) and 96% (78, 100), respectively. In Cohort B, 22% (28/129) of people with a contaminated culture were Xpert MTB/XDR TB-positive. Of these, 57% (16/28), 7% (2/28), and 43% (12/28) were isoniazid-, fluoroquinolone-, and ethionamide-resistant (in 2, 1, and 4 people respectively, this would be the first resistant result). In both cohorts, time-to-DST could improve a median (IQR) of 22 (12–42) days.

**Conclusion:**

Xpert MTB/XDR on contaminated MGIT960 cultures had high sensitivity and specificity for DST. This approach could mitigate culture contamination’s negative effects and improve gaps in the drug-resistant TB diagnostic cascade.

## Introduction

Tuberculosis (TB) remains a global health crisis, with 8.2 million new cases and 1.3 million deaths in 2023. In 2023, an estimated 400,000 rifampicin-resistant cases occurred, but 22% were never diagnosed^1^. Furthermore, drug-resistant diagnoses fell by 15% between 2019 and 2022 due to COVID-19^1^. Drug-resistant TB diagnosis requires improvement.

In addition to better upfront diagnosis of first-line resistance, a key drug-resistant TB care cascade gap is the many people with first-line resistance who do not get second-line drug susceptibility testing (DST). In South Africa in 2023, 12% of multidrug-resistant/rifampicin-resistant (MDR/RR)-TB cases did not have second-line DST attempted^1^. Furthermore, in those with it attempted, second-line DST was successful (a resistant or susceptible result generated for a second-line drug) in only 66% of people. Due to these unsuccessful results, which can be frequent from paucibacillary sputum, national algorithms often culture sputum in parallel to direct molecular testing (the isolate may be used for repeat molecular testing and/or phenotypic DST). During culture, contamination can occur and, even if people do return to provide another specimen, care cascade delays and loss occurs.

These missed opportunities for second-line DST due to contamination are especially important given the advent of new and repurposed lifesaving Group A drugs^2^. Although rapid tests for resistance to these drugs are for the most part not yet widely available, resistance is more likely to emerge if the number of effective drugs is low^3^. Maximising second-line DST, including by ensuring people with contaminated culture receive appropriate DST as early as possible, is therefore important to protect Group A drugs.

Xpert MTB/XDR (Cepheid, Sunnyvale, USA) is a cartridge-based real-time PCR test endorsed by WHO in 2021 as a low complexity reflex test for second-line DST in people with MDR/RR-TB^4^. Xpert MTB/XDR must detect *Mycobacterium tuberculosis* complex (MTBC) DNA before it can evaluate whether variants associated with resistance to isoniazid, ethionamide, fluoroquinolone, and second-line injectable drugs (SLIDs) such as kanamycin, amikacin, and capreomycin are present. Results are automatically interpreted by GeneXpert software^5^.

Xpert MTB/XDR is validated on sputum (raw and processed) and culture isolates^6^. It has not evaluated on contaminated Mycobacterium Growth Indicator Tube (MGIT960, Becton Dickinson Systems, USA) cultures. Contaminated cultures, which occurs typically at 5–8% in South Africa, are routinely not further processed and are discarded, prompting a request for a repeat specimen submission for culture^7^. However, high rates of repeat specimen non-submission (49%) have been reported in South Africa^7^. Furthermore, the median turnaround-time (TAT) for sputum resubmission, if it occurs, is 42 days. Lastly, it is possible that people who may stand to benefit the most from second-line DST are at elevated risk of culture contamination (due to, for example, microbiome perturbations due to prior antibiotic use from failing regimens, less fit strains due to drug resistance are more prone to overgrowth by contaminants, clinics in underserved high burden communities less able to adhere to good specimen collection practice).

We have shown promising accuracy results (high sensitivity and specificity) when applying the Xpert MTB/RIF Ultra (Ultra, Cepheid, Sunnyvale, USA) on contaminated cultures in the presence of non-mycobacterial contaminants for the detection of TB and rifampicin resistance^7^. The current study aims to evaluate diagnostic performance and potential effect (TAT) of Xpert MTB/XDR on contaminated MGIT960 growth from people with Xpert MTB/RIF Ultra-diagnosed MDR/RR-TB for drug resistance.

## Methods

### Study design and setting

#### Routine specimen processing and programmatic MGIT960 culture

**Figure 1:**
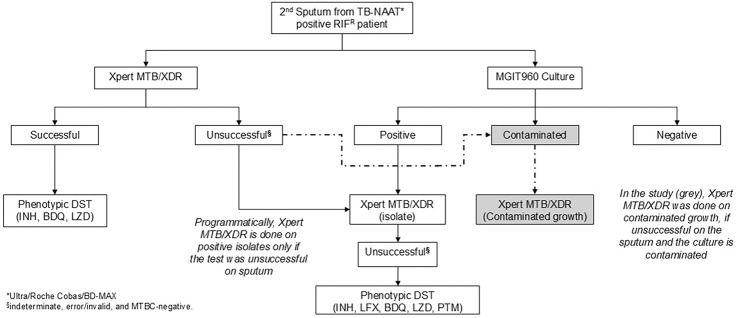
A routine DST algorithm with Xpert MTB/XDR on contaminated cultures added as done in the study (grey, dot-dash line). Abbreviations: BDQ, bedaquiline; DST, drug susceptibility testing; INH, isoniazid, LFX, levofloxacin; LZD, linezolid; MGIT960, Mycobacterium growth indicator tube 960; MTBC, *Mycobacterium tuberculosis*complex; PTM, pretomanid; TB-NAAT, tuberculosis nucleic-acid amplification testing.

In this retrospective diagnostic accuracy study, we included people who submitted sputa to the National Health Laboratory Service (NHLS) Greenpoint, Cape Town, South Africa. MGIT960 culture from diagnostic sputum is performed when the Ultra (the initial diagnostic test for individuals with presumptive TB) rifampicin susceptibility result is resistant or indeterminate, or for monitoring treatment response (Fig. 1, **Supplementary methods**). For initial diagnosis, sputum from people with MDR/RR-TB is used for Xpert MTB/XDR testing in parallel to culture. If Xpert MTB/XDR is unsuccessful, it is re-attempted on the isolate in people who have AFB-positive growth. For treatment monitoring, Xpert MTB/XDR is not done on sputum and only AFB-positive growth.

##### Sample and contaminated culture selection:

**Figure 2:**
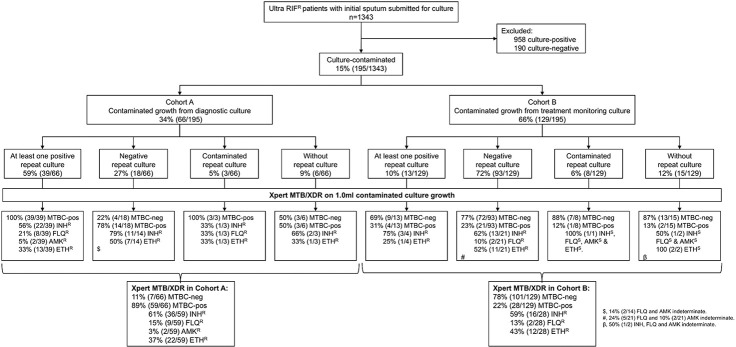
Study profile. We quantified cultures done on Xpert Ultra rifampicin-resistant respiratory specimens in a high-volume programmatic laboratory. Our approach revealed resistance in 52 cases for isoniazid, 11 for fluoroquinolone, two for amikacin, and 34 for ethionamide that were missed or delayed and could likely be contributing to transmission. Xpert MTB/XDR, when done on AFB-negative contaminated growth, is concordant with culture and susceptibility results from a repeat specimen. Abbreviations: AMK^R^, amikacin-resistant; ETH^R^, ethionamide-resistant; FLQ^R^, fluoroquinolones-resistant; IND, indeterminate; INH^R^, isoniazid-resistant; MTBC, *Mycobacterium tuberculosis* complex; Ultra, Xpert MTB/RIF Ultra.

Contaminated AFB-negative cultures from people with MDR/RR-TB were consecutively collected from 01 October 2023 to 31 March 2024, with samples stored at 2–8°C. Contaminated cultures were either submitted for diagnosis (from Cohort A; culture done as Xpert MTB/XDR on sputum unsuccessful) or treatment monitoring (Cohort B; culture done to check for positivity and, if positive, resistance). The Cohorts were mutually exclusive. Results of routine TB investigations on specimens or isolates up to three months after initial contamination detection were captured [Ultra, culture, Xpert MTB/XDR, phenotypic DST (pDST)]. Eligible people were further categorised based on their repeat culture results into four subsets: repeat-culture positives, repeat-culture negatives, repeat-culture contaminated, and those without any repeat culture attempted (Fig. 2).

### Procedures

#### Xpert MTB/XDR

Briefly, 2.0 ml of sample reagent (Cepheid, Sunnyvale, CA, USA) was added to 1.0 ml of contaminated culture, and the sample processed per the manufacturer’s instructions^6^.

#### Reference standard

For TB, people were classified as definite TB if at least one subsequent culture was TB-positive within the three-month follow-up period. If a person only had MTBC-negative repeat results, they were designated TB-negative. For DST, resistant cases had definite TB and resistance on a subsequent specimen or isolate within the follow-up period from Xpert MTB/XDR, and/or pDST; reference standard susceptible cases were definite TB and had only susceptible result(s).

### Statistical analysis and definitions

Xpert MTB/XDR sensitivity and specificity for TB and DST were estimated using 2×2 tables, with 95% confidence intervals (CIs) calculated via the exact binomial method and Stata (version 18, StataCorp). Diagnostic delay from culture contamination was defined as the days between the initial contamination report date and the earliest subsequent result for TB or DST from a repeat specimen. The date of the earliest subsequent culture-negative result was used for patients with repeat specimens that yielded no positive results. Potential TAT improvements were calculated by comparing diagnostic delay to if Xpert MTB/XDR was done on contaminated growth the day after contamination detection.

### Ethics statement

This study received approval from the Human Research Ethics Committee Division of Molecular and Human Genetics, Department of Biomedical Sciences at Stellenbosch University (S20/08/189) and the NHLS (PR2119347). Permission was granted to access anonymised to-be-discarded residual samples collected as part of routine diagnostic practice with informed consent waived.

## Results

### Quantity of routine diagnostic cultures

1343 people had Ultra rifampicin-resistant sputum; 48% (650/1343) in Cohort A and 52% (693/1343) in Cohort B. Overall, 71% (958/1343) were culture-positive, 14% (190/1343) culture-negative, and 15% (195/1343) culture-contaminated (66 Cohort A, 129 Cohort B) (Fig. 2). The median age was 37 years (IQR: 30–45), 65% (126/195) of people were men, 65% living with HIV (108/165; 30 unknown HIV status), and 49% had prior TB (94/191; 4 unknown prior TB status).

### Cohort A (contaminated cultures at initial diagnosis)

9% (6/66) of people had no repeat specimens. Of those with repeats, 65% (39/60) had at least one repeat specimen culture-positive, 30% (18/60) had all repeat specimen(s) culture-negative [78% (14/18) were Xpert MTB/XDR TB-positive)], and 5% (3/60) had only culture-contaminated results for repeats. For TB detection, contaminated growth from 57 (39 plus 18) people hence existed with reference standard information and, for DST, 39 people. 27 (6 plus 18 plus 3) people had no further DST information.

### Cohort B (contaminated cultures during treatment monitoring)

12% (15/129) of people had no repeat specimens. Of those with repeats, 11% (13/114) had at least one repeat specimen culture-positive, 82% (93/114) had all repeat specimen(s) culture-negative, and 7% (8/114) had only culture-contaminated repeat result(s). For TB detection, contaminated growth from 106 (13 plus 93) people hence existed with reference standard information and, for DST, 3 people. 126 (15 plus 93 plus 8 plus 10) people have no further DST information.

### Xpert MTB/XDR diagnostic accuracy on contaminated cultures

#### MTBC-detection:

**Figure 3:**
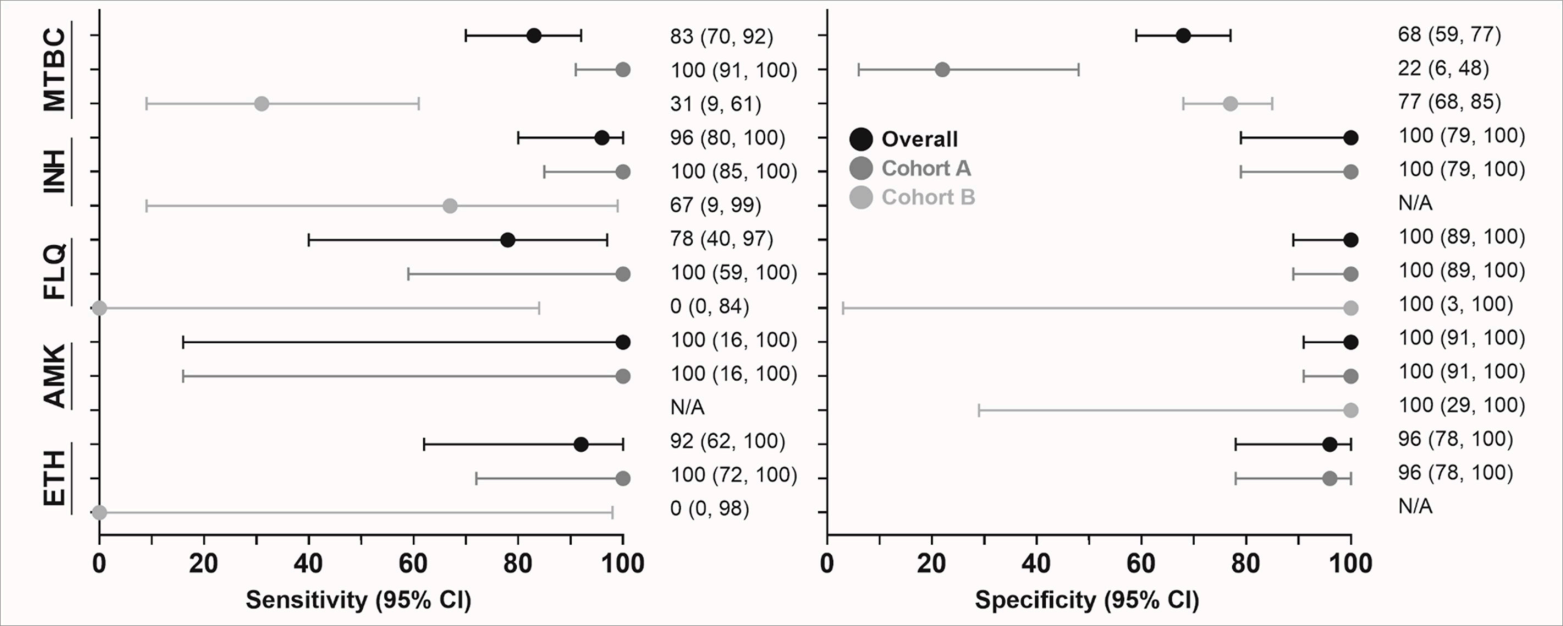
Forest plot of sensitivity and specificity (with 95% confidence intervals) for Xpert MTB/XDR on contaminated cultures. Data are given overall and per Cohort. As expected, MTBC sensitivity in people on treatment was less than diagnostic samples (Cohorts B vs. A). Sensitivity for resistance is like that reported for Xpert MTB/XDR on sputum, and specificity generally excellent, suggesting that a Xpert MTB/XDR resistance result from a contaminated culture can be trusted. Cohort B has limited reference standard data for FLQ, AMK and ETH. The number of people used to calculate each estimate is in **Supplementary Table 1**. Abbreviations: AMK, amikacin; CI, confidence interval; ETH, ethionamide; FLQ, fluoroquinolones; INH, isoniazid; MTBC, *Mycobacterium tuberculosis*complex.

Of 163 people (57 Cohort A, 106 Cohort B) with reference standard information, 48% (78/163) were TB-positive and 52% (85/163) MTBC-negative by Xpert MTB/XDR on contaminated growth. Sensitivity and specificity were 83% (95% CI: 70, 92) and 68% (59, 77), respectively (**Supplementary Fig. 1**). In Cohort A, sensitivity and specificity was 100% (91, 100) and 22% (6, 48), and in Cohort B 31% (9, 61) and 77% (68, 85), respectively (Fig. 3). Aggregate data (both Cohorts) for MTBC and DST are on **Supplement pg. 3**.

#### Isoniazid susceptibility

42 people had reference standard information (39 Cohort A, 3 Cohort B). Sensitivity and specificity were 96% (80, 100) and 100% (79, 100), respectively. In Cohort A, sensitivity was 100% (85, 100) and specificity 100% (79, 100). Too few Cohort B people had reference standard information to calculate sensitivity and specificity (all three reference standard resistant), but two were detected as resistant and one false-susceptible by contaminated growth Xpert MTB/XDR.

#### Fluoroquinolones susceptibility

41 people had reference standard information (38 Cohort A, 3 Cohort B). Sensitivity and specificity were 78% (40, 97) and 100% (89,100), respectively. In Cohort A, sensitivity was 100% (59, 100) and specificity 100% (89, 100). In Cohort B, two-reference standard resistant people were Xpert MTB/XDR false-susceptible and one correctly detected as susceptible.

#### Amikacin susceptibility

42 people had reference standard information (39 Cohort A, 3 Cohort B). Sensitivity and specificity were 100% (16, 100) and 100% (91, 100), respectively. In Cohort A, sensitivity was 100% (16, 100) and specificity 100% (91, 100). In Cohort B, all three were reference standard susceptible and correctly detected.

#### Ethionamide susceptibility

35 people had reference standard information (34 Cohort A, 1 Cohort B). Sensitivity and specificity were 92% (62, 100) and 96% (78, 100), respectively. In Cohort A, sensitivity was 100% (72, 100) and specificity 96% (78, 100). In Cohort B, the reference standard resistance person was Xpert MTB/XDR false-susceptible.

### Xpert MTB/XDR results in people without reference standard results

#### Cohort A

Of the 14% (9/66) that did not have reference standard results (6 without repeat submission, 3 repeat culture-contaminated), 67% (6/9) were Xpert MTB/XDR TB-positive and three of these had resistance to at least one drug detected by Xpert MTB/XDR on contaminated growth (n = 1 isoniazid-resistant; n = 1 isoniazid-,fluoroquinolone-, and ethionamide-resistant; n = 1 isoniazid- and ethionamide-resistant).

#### Cohort B

Of the 18% (23/129) that did not have reference standard results, 13% (3/23) were TB-positive. Of these, two had no Xpert MTB/XDR-detected resistance on contaminated growth, and one was ethionamide-susceptible and isoniazid-, fluoroquinolone-, amikacin-indeterminate.

### Incremental yield in resistance detection if contaminated Xpert MTB/XDR approach used

In the study period, 90% (1148/1343) people had a routine Xpert MTB/XDR on a sputum or isolate that successfully generated a resistant or susceptible result to at least one drug: 1141, 1132, 1132, and 1147 for isoniazid, fluoroquinolones, amikacin, and ethionamide, respectively (after removal of indeterminates). For each drug, 522, 93, 45, and 335, respectively were Xpert MTB/XDR-resistant. Amongst the 195 people with contaminated culture growth, Xpert MTB/XDR on this growth successfully generated a resistant or susceptible result in 86, 79, 82, and 87 people for each drug, respectively (52, 11, 2, and 34 resistant). The people who successfully received DST therefore increased by 8% (6, 9), 7% (6, 9), 7% (6, 9), and 8% (6, 9), respectively and resistance diagnosed in the study period would, should the contaminated Xpert MTB/XDR approach be used, have increased by 10% (8, 13), 12% (6, 20), 4% (1, 15), and 10% (7, 14) for isoniazid, fluoroquinolones, amikacin, and ethionamide, respectively (Table 1). In Cohort A, all this additionally diagnosed resistance was resistance hitherto unknown to the programme.

### Cohort B resistance documented for the first time

Of the 22% (28/129) people Xpert MTB/XDR TB-positive, 21% (6/28) had resistance to a specific drug detected for the first time using the contaminated Xpert MTB/XDR approach. Of these, one had newly diagnosed isoniazid and ethionamide resistance, and one newly diagnosed isoniazid-resistance, one newly diagnosed fluoroquinolone-resistance, and three newly diagnosed ethionamide-resistance (these three, which were also isoniazid resistant, had earlier documented isoniazid resistance).

### Potential turnaround-time improvements

The overall potential improved TAT for DST was a median (IQR) of 22 (12–42) days in Cohort A and in Cohort B 28 (12–86) days.

## Discussion

We describe the feasibility of Xpert MTB/XDR on contaminated cultures. Key findings are: 1) sensitivity to detect TB (100% in Cohort A), which is required for Xpert MTB/XDR to generate DST results, is comparable to that reported^5^ on sputum and 2) isoniazid-, fluoroquinolone-, amikacin-, and ethionamide-resistance detection. 3) Our approach could increase people diagnosed with resistance (12% for fluoroquinolones in our setting), reduce delays and costs associated with the need to collect and culture a second specimen, and generate a DST result in patients who had none before (importantly, many patients had no repeat specimens, meaning the contaminated Xpert MTB/XDR approach would be the only resistant result). Lastly, 4) our approach identified a high proportion of rifampicin-resistant people who were isoniazid-susceptible that, in the event of contamination, could be deprived or delayed initiation of an isoniazid-containing second-line regimen. Together, these findings have implications for reducing laboratory care cascade loss.

Our approach of applying Xpert MTB/XDR on contaminated culture growth initially inoculated because the initial sputum Xpert MTB/XDR was negative or unsuccessful had similar sensitivity for TB as that reported on sputum^5,8^ and all people whose later culture was positive were contaminated growth Xpert MTB/XDR TB-positive. Our previous work using Ultra on contaminated cultures also found that sensitivity for TB did not differ to that on sputum^7^. This is important because, with any diminished ability of Xpert MTB/XDR to detect TB on contaminated growth, fewer susceptibility results will be reported. In other words, this finding establishes feasibility.

Xpert MTB/XDR has suboptimal specificity for TB (78% of repeat culture-negative people were Xpert MTB/XDR TB-positive) but this is inconsequential because these people are already known to have TB and is expected due to treatment between the collection of the two specimens (during which culturability decreases quicker than DNA positivity^9^). The detection of contaminated culture resistance by Xpert MTB/XDR could, even in people whose next culture result is negative, indicate early detection of resistance in bacilli with diminished culturability^10,11^, especially if this resistance was previously not detected.

Sensitivities and specificities for isoniazid-, fluoroquinolone-, amikacin-, and ethionamide-susceptibility on contaminated cultures were comparable to those on sputa and isolates^5^, with 100% sensitivity for all drugs except fluoroquinolone (one discordant result). The discordant fluoroquinolone result was contaminated culture Xpert MTB/XDR susceptible, but the reference standard resistant on an isolate collected 87 days later than the sputum that was initially culture contaminated. This may be because of treatment, leading to a selection of an initially hetero-resistant sub-population^10,11^.

Adding Xpert MTB/XDR on contaminated cultures to diagnostic algorithms would lead to both more people receiving a molecular test for second-line resistance and a higher diagnostic yield. Importantly, even though in our setting a minority of people eligible for Xpert MTB/XDR on the sputum or isolate did not programmatically receive a Xpert MTB/XDR resistant or susceptible result due to culture contamination, we show that if our approach were applied, 4–12% more resistance (drug dependent x) would be identified. This would be associated with improved DST and most beneficial in people where a repeat specimen was not submitted the and who never receive second-line DST. Such DST improvements can help TB programmes meet targets to find undiagnosed resistance, a WHO EndTB priority^12^. This is especially applicable to settings that, unlike ours, have contamination rates more than the “acceptable range” of 3–8%^13^ (15–30% has been reported^7^) and therefore experience higher drop out or delays of people eligible for Xpert MTB/XDR testing on culture isolates. Such environments would benefit the most from this approach.

In addition to second-line drug resistance, our approach confirmed a surprisingly high rate of isoniazid susceptibility (40%) in people already diagnosed as rifampicin-resistant. Rifampicin-resistant people are typically defaulted to a second-line regimen and, in the event of culture required to do isoniazid DST, would be deprived of rapid inclusion of isoniazid in their regimen if culture was contaminated. This finding supports other studies challenging the assumption that rifampicin-resistance indicates MDR-TB^14,15^, highlighting the potential benefit of Xpert MTB/XDR.

The results should be interpreted in the context of limitations. Xpert MTB/XDR was done on a different specimen than the reference standard, leading to a possible discordance due to varying bacilli concentration and effects of treatment on strains, but we operated under the assumptions that a Xpert MTB/XDR DST result was better than none and that, should a second specimen be forthcoming, Xpert MTB/XDR retesting of sputum or a culture-positive isolate could always be re-attempted. Our study was also pragmatic and some people had missing or limited DST data from the lack of follow-up cultures, however, this highlights the need for innovative approaches to addressing gaps in the routine diagnostic pathways^16,17^.

In conclusion, Xpert MTB/XDR on contaminated cultures is accurate in diagnosing TB and isoniazid-, fluoroquinolone-, amikacin- and ethionamide-resistance. Our approach allows for reduced cascade loss, and improved DST TAT. Diagnostic algorithms may consider implementing this approach in laboratories that experience contamination when attempting DST beyond rifampicin resistance.

## Figures and Tables

**Table 1:** Incremental yield. Number tested by Xpert MTB/XDR programmatically in the study period (on sputum and/or isolates), the number of people in that period in whom this Xpert MTB/XDR could not be done due to contamination, and yield increase in people who had a Xpert MTB/XDR result when the contaminated Xpert MTB/XDR approach was used (and the resulting increase in resistance diagnoses).

Drugs	Number programmatically Xpert MTB/XDR resistant (of the 1148 tested)	Number in whom Xpert MTB/XDR could not be done programmatically because it was unsuccessful on sputum, culture was contaminated, and Xpert MTB/XDR detected resistance on the contaminated growth (of the 195 tested)	Proportion increases in Xpert MTB/XDR-detected resistance % (95% CI)
INH	522	52	10 (8, 13)
FLQ	93	11	12 (6, 20)
AMK	45	2	4 (1, 15)
ETH	335	34	10 (7, 14)

Abbreviations: AMK, amikacin; CI, confidence interval; ETH, ethionamide; FLQ, fl uoroquinolones; INH, isoniazid.

## Data Availability

Study data can be accessed on request from the corresponding author without restriction.
